# Next generation sequencing of exceptional responders with *BRAF*-mutant melanoma: implications for sensitivity and resistance

**DOI:** 10.1186/s12885-015-1029-z

**Published:** 2015-02-18

**Authors:** Jennifer Wheler, Roman Yelensky, Gerald Falchook, Kevin B Kim, Patrick Hwu, Apostolia M Tsimberidou, Philip J Stephens, David Hong, Maureen T Cronin, Razelle Kurzrock

**Affiliations:** 1 Department of Investigational Cancer Therapeutics – a Phase I Clinical Trials Program, Unit 455, The University of Texas MD Anderson Cancer Center, 1515 Holcombe Blvd, Houston, TX 77030 USA; 2 Foundation Medicine, 150 Second Street, Cambridge, MA 02141 USA; 3 Department of Melanoma Medical Oncology, The University of Texas MD Anderson Cancer Center, 1515 Holcombe Blvd, Houston, TX 77030 USA; 4 Center for Personalized Cancer Therapy, Moores Cancer Center, The University of California San Diego, 3855 Health Sciences Drive, La Jolla, CA 92093 USA

**Keywords:** *BRAF* mutation, Melanoma, Next generation sequencing, Resistance, Time to treatment failure

## Abstract

**Background:**

Patients with *BRAF* mutation-positive advanced melanoma respond well to matched therapy with BRAF or MEK inhibitors, but often quickly develop resistance.

**Methods:**

Tumor tissue from ten patients with advanced *BRAF* mutation-positive melanoma who achieved partial response (PR) or complete response (CR) on BRAF and/or MEK inhibitors was analyzed using next generation sequencing (NGS) assay. Genomic libraries were captured for 3230 exons in 182 cancer-related genes plus 37 introns from 14 genes often rearranged in cancer and sequenced to average median depth of 734X with 99% of bases covered >100X.

**Results:**

Three of the ten patients (median number of prior therapies = 2) attained prolonged CR (duration = 23.6+ to 28.7+ months); seven patients achieved either a PR or a short-lived CR. One patient who achieved CR ongoing at 28.7+ months and had tissue available close to the time of initiating BRAF inhibitor therapy had only a BRAF mutation. Abnormalities in addition to *BRAF* mutation found in other patients included: mutations in *NRAS, APC* and *NF1*; amplifications in *BRAF*, aurora kinase A, *MYC, MITF* and *MET*; deletions in *CDKN2A/B* and *PAX5*; and, alterations in *RB1* and *ATM*. Heterogeneity between patients and molecular evolution within patients was noted.

**Conclusion:**

NGS identified potentially actionable DNA alterations that could account for resistance in patients with *BRAF* mutation-positive advanced melanoma who achieved a PR or CR but whose tumors later progressed. A subset of patients with advanced melanoma may harbor only a *BRAF* mutation and achieve a durable CR on *BRAF* pathway inhibitors.

**Electronic supplementary material:**

The online version of this article (doi:10.1186/s12885-015-1029-z) contains supplementary material, which is available to authorized users.

## Background

Over 50% of melanomas are characterized by the presence of a *BRAF* mutation [1]. The most common *BRAF* mutation (*BRAF* V600E) leads to constitutive activation of the mitogen-activated protein kinase (MAPK) pathway. Targeting BRAF with RAF-selective inhibitors has demonstrated remarkable tumor shrinkage in those tumors with *BRAF* mutations [2-4]. Despite these remarkable results, response to BRAF inhibitors is transient for most patients with advanced melanoma.

Previous pre-clinical studies have shown that retreatment with a second BRAF inhibitor in cells that have become resistant to another BRAF inhibitor is unlikely to be an effective strategy [5]. Nor are secondary *BRAF* mutations believed to play a large role after development of resistance [6,7]. However, reactivation of MAPK pathway through various mechanisms may be in part responsible for the development of acquired resistance [6]. Combination strategies may help to overcome resistance. For example, combining a BRAF inhibitor with agents that target insulin-like growth factor 1 receptor (IGF1R), downstream phosphatidylinositol 3-kinase (PI3K)/AKT signaling and/or MEK pathways may serve to enhance therapeutic effects [5,8]. Identification of molecular alterations in addition to *BRAF* may help explain why resistance develops more quickly in some patients, and suggest rationale strategies to overcome resistance.

In this pilot study, we investigated patients with advanced melanoma who were responders on clinical trials using BRAF and/or MEK inhibitors.

## Methods

### Patients

Patients with *BRAF*-mutant, advanced melanoma who experienced treatment failure with standard therapy, and subsequent partial response (PR) or complete response (CR) on BRAF, MEK, and BRAF/MEK combination targeted trials, and who had tissue available for molecular analysis were eligible. The study was carried out by collaboration between the Department of Investigational Cancer Therapeutics (Phase I Clinical Trials Program) at The University of Texas MD Anderson Cancer Center (MD Anderson) and Foundation Medicine (Boston, MA). The registration of patients in the database, pathology assessment, and preliminary limited mutation analysis (see below) were performed at MD Anderson. Subsequent molecular evaluation with NGS was performed at Foundation Medicine. The study was reviewed and approved by the MD Anderson Institutional Review Board (IRB 5 IRB00006023) with a waiver of authorization to use and disclose protected health information. All patients consented for experimental therapeutic interventions according to institutional guidelines, and all patients had consented to anonymized assessments and analysis of data and outcome of therapy.

### Tissue samples and molecular analyses

Mutation analysis at MD Anderson: *A*rchival formalin-fixed, paraffin-embedded tissue blocks or material from fine-needle aspiration biopsy obtained from diagnostic and/or therapeutic procedures was used to test for *BRAF* mutations. *BRAF* mutation testing was performed in the CLIA–certified Molecular Diagnostic Laboratory within the Division of Pathology and Laboratory Medicine at MD Anderson. DNA was extracted from micro-dissected paraffin-embedded tumor sections and analyzed using a polymerase chain reaction (PCR)-based DNA sequencing method for *BRAF* codons 595–600 mutations of exon 15 by pyrosequencing as previously described [9]. Whenever possible, testing for other mutations such as Kirsten rat sarcoma viral oncogene homolog (*KRAS*) and neuroblastoma rat sarcoma viral oncogene homolog (*NRAS*), *PIK3CA* [10], and *TP53* was performed. Phosphatase and tensin homolog (PTEN) deletion was assessed using immunohistochemistry and the DAKO antibody (Carpinteria, Ca.) [11].

NGS analysis at Foundation Medicine: Genomic libraries were captured for 3230 exons in 182 cancer-related genes plus 37 introns from 14 genes often rearranged in cancer and sequenced to average median depth of 734X with 99% of bases covered >100X [12] (Additional file 1). The molecular alterations were reported as somatic alterations of known significance and somatic alterations of unclear significance based on the impact of these molecular alterations on tumorigenesis as stated in the scientific literature.

### Treatment and evaluation

Starting in July 2010, consecutive patients with melanoma and available tissue who achieved a PR or CR while on a BRAF and/or MEK inhibitor were studied. Treatment continued until disease progression or unacceptable toxicity occurred.

Assessments were performed as specified in each protocol at the beginning of each new treatment cycle. Efficacy was assessed using computed tomography and/or positron emission tomography scan at baseline and then every two cycles (eight weeks). All radiographs were read in the Department of Radiology at MD Anderson. Responses were categorized per Response Evaluation Criteria in Solid Tumors (RECIST) version 1.0 or 1.1 depending on the study the patient was enrolled in (Additional file 2) and were reported as best response [13,14].

### Statistical analysis

This is a pilot study with descriptive analyses used to summarize patient characteristics. Time to treatment failure (TTF) was defined as the time interval from the start of therapy to the first observation of disease progression per RECIST version 1.0 or 1.1 depending on the study, or death, or removal from study for any reason.

## Results

### Patients

A total of ten patients with *BRAF*-mutant, advanced melanoma who had achieved a PR or CR on a BRAF and/or MEK targeted drug were analyzed for molecular alterations. The median age was 52 years (range, 23 to 60 years) and all ten patients were Caucasian (100%). These patients had received a median of two prior therapies in the metastatic setting. Five patients had an Eastern Cooperative Oncology Group (ECOG) performance status [15] of 0 and five patients had an ECOG of 1. The patient characteristics are summarized in Table 1.

**Table 1:** **Demographics and clinical characteristics of 10 patients with** ***BRAF*** **mutation- positive melanoma**

Variable	Group	No. of patients	%
		(*n =* 10)	
Age, years	Median	52	
	Range	23-60	
	<50	5	50
	≥50	5	50
Sex	Men	7	70
	Women	3	30
Race	Caucasian	10	100
Number of prior therapies	Median	2	
	Range	0-5	
	≤2	8	80
	>2	2	20
Lactate dehydrogenase^a^	≤upper limit of normal	9	90
	>upper limit of normal	1	10
TNM stage M1c	No	6	60
	Yes	4	40
ECOG PS	0	5	50
	1	5	50
Therapy	BRAF inhibitor alone	7	70
	MEK inhibitor alone	1	10
	BRAF/MEK inhibitor combination	2	20
Treatment response	Complete response	4	40
	Partial response	6	60

^a^Upper limit of normal in our institution = 618 IU/L.

*Abbreviations:ECOG* Eastern Cooperative Oncology Group, *PS* Performance Status, *TNM* tumor, node, metastasis.

**Figure 1:**
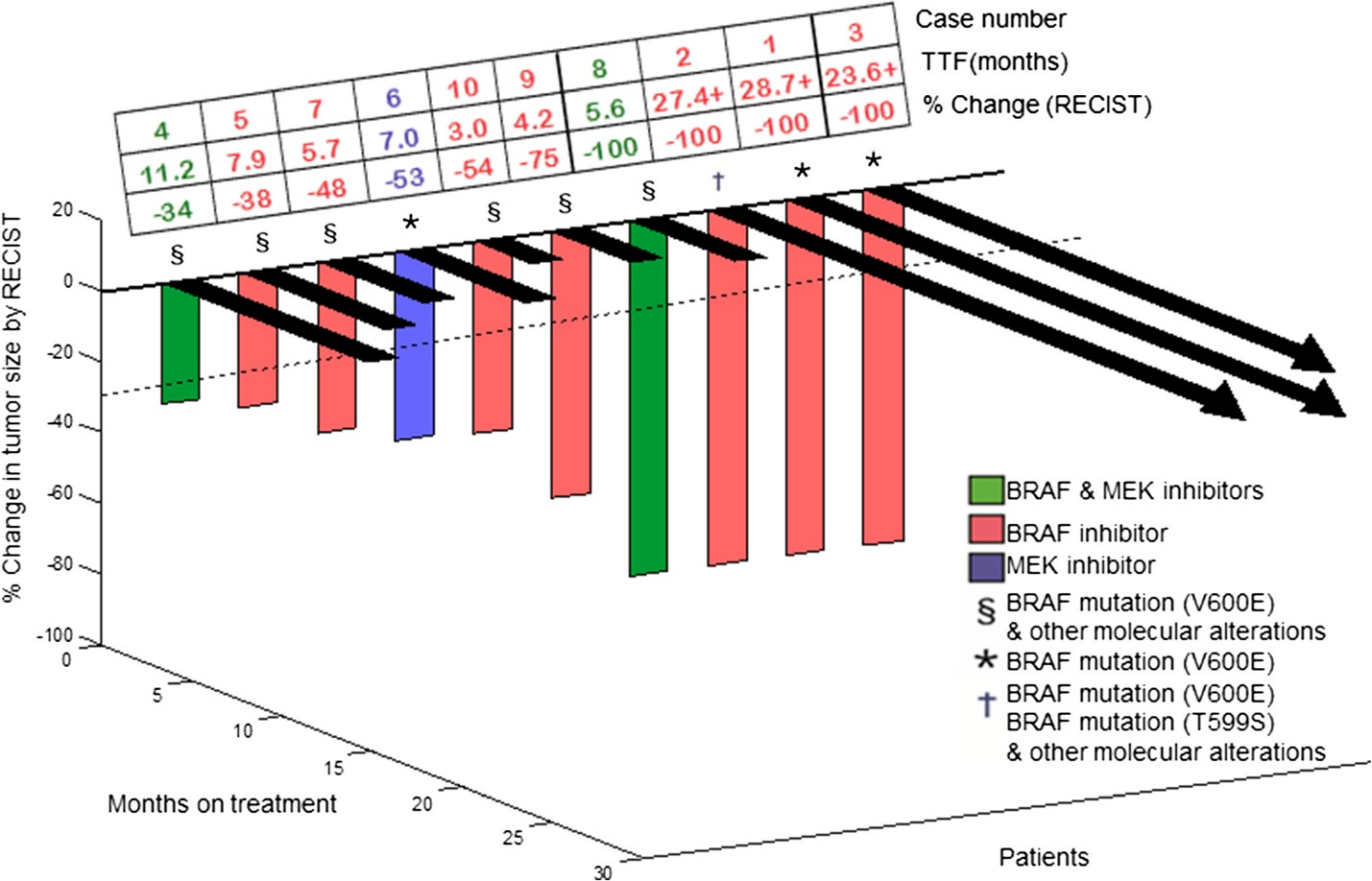
**3-D waterfall plot.** Best response by RECIST, of ten patients with *BRAF-*positive melanoma. Time to treatment failure in months is represented by solid lines and the arrow indicates that the patient was still on study when the data was censored.

**Figure 2:**
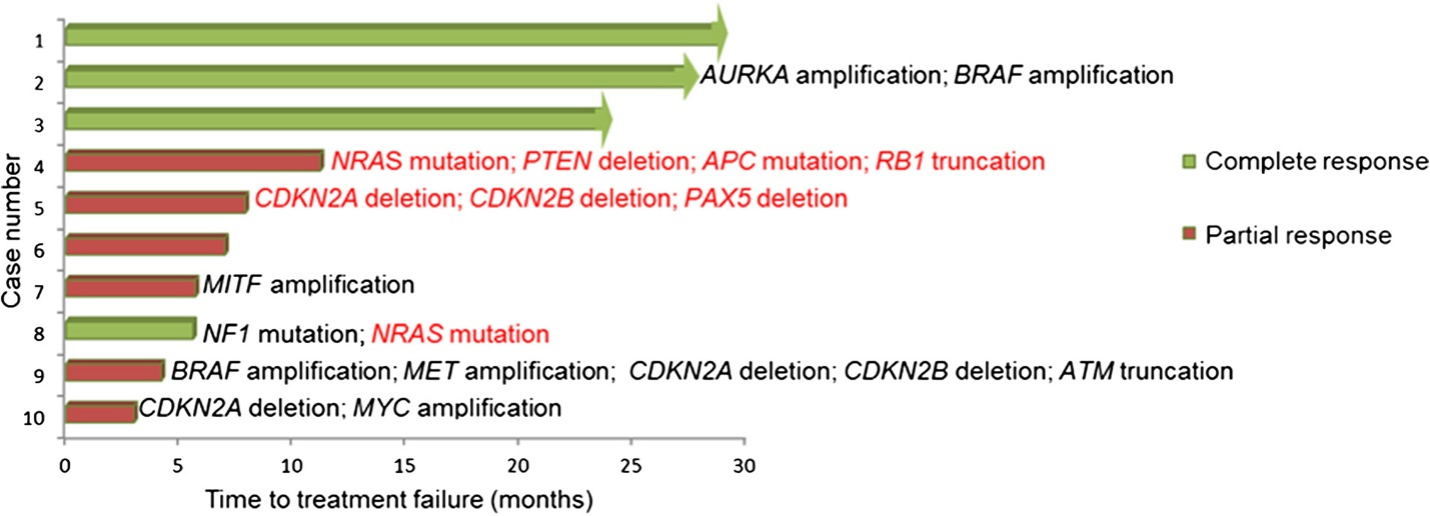
**Time- to-treatment failure and additional molecular alterations in ten patients with*BRAF*-mutant melanoma.** Arrow indicates that the patient was still on study when the data was censored. A comprehensive list of alterations and their timing is found in Table 2 and Additional file 3. Red text refers to alterations seen in post-treatment biopsies.

Nine of ten patients with *BRAF* mutations were treated with BRAF inhibitors, either as a single agent (7 patients) or in combination with a MEK inhibitor (2 patients). The remaining one patient was treated with a single-agent MEK inhibitor. The clinical trials are summarized in Additional file 2. Four of ten patients (40%) had a CR, three of which are ongoing for about two years or longer (TTF = 5.6, 23.6+, 27.4+, and 28.7+ months). Six patients (60%) attained a PR (TTF = 3.0, 4.2, 5.7, 7.0, 7.9, and 11.2 months) (Figures 1 and 2).

### Somatic genomic alterations

Eight of ten patients had results of NGS analysis performed on tissues biopsied prior to treatment (range, 2 to 53 months) (Table 2). In two patients, NGS analyses were done on tissues biopsied after treatment initiation; in one case (case #4), the tissue for NGS analysis was obtained six months after treatment initiation from a continuously progressive lesion; in another case (case #5), NGS analyses was performed on three different tissue samples from progressive lesions (at 6, 9 and 26 months after treatment initiation).

**Table 2:** **NGS-based molecular alterations in** ***BRAF*** **-positive melanoma responders**

Case no.	*BRAF*mutation	Type of targeted drug	Treatment start date	Best response (%)	TTF (months)^a^	Sampling date; time from treatment^b^	Molecular analysis by NGS		
							Mutations	Amplifications/deletions	Truncations
1	V600E	BRAF inhibitor	9/17/2009	CR (−100)	28.7+	5/1/2009; −4 months	BRAF (V600E)	None	None
2	V600E	BRAF inhibitor	10/7/2009	CR (−100)	27.4+	1/22/2009; −9 months	BRAF (V600E); BRAF (T599S)	AURKA amplification; BRAF amplification	None
	T599S								
3	V600E	BRAF inhibitor	1/27/2010	CR (−100)	23.6+	8/19/2005; −53 months	None^c^	None	None
4	V600E	BRAF + MEK inhibitor	11/11/2010	PR (−34)	11.2	5/17/2011; +6 months	APC (R1171H); BRAF (V600E); NRAS (Q61R)	PTEN deletion	RB1 truncation
5	V600E	BRAF inhibitor	6/17/2009	PR (−38)	7.9	12/2/2009; +6 months	BRAF (V600E)	CDKN2A deletion; CDKN2B deletion; PAX5 deletion	None
						3/23/2010; +9 months	BRAF (V600E)	CDKN2A deletion; CDKN2B deletion; PAX5 deletion	NF1 truncation
						8/24/2011; +26 months	None	None	None
6	V600E	MEK inhibitor	4/1/2010	PR (−53)	7.0	9/17/2007; −31 months	BRAF (V600E)	None	None
7	V600E	BRAF inhibitor	1/12/2010	PR (−48)	5.7	11/5/2009; −2 months	BRAF (V600E)	MITF amplification	None
8	V600E	BRAF + MEK inhibitor	9/3/2010	CR (−100)	5.6	4/7/2009; −17 months	BRAF (V600E); NF1 (R 440*)^d^	None	None
9	V600E	BRAF inhibitor	1/27/2010	PR (−75)	4.2	11/11/2009; −2 months	BRAF (V600E)	BRAF amplification; MET amplification; CDKN2A deletion; CDKN2B deletion	ATM truncation
10	V600E	BRAF inhibitor	4/15/2010	PR (−54)	3.0	12/11/2009; −4 months	BRAF (V600E)	MYC amplification; CDKN2A deletion	None

^a^ ‘+’ = continuing on the study when data was censored.

^b^ ‘ + ’ = number of months biopsy was taken after treatment; '-' = number of months biopsy preceded treatment.

^c^ = tissue sample obtained on 10/29/2008 was *BRAF* mutation-positive (V600E) by single PCR assay (case #3).

^d^ = tissue sample obtained from a progressive hepatic lesion on 12/27/2010 had an *NRAS* mutation (Q61K) in codon 61 by single PCR assay (case #8).

*Abbreviations:CR* Complete response, *NGS* Next generation sequencing, *PR* Partial response, *TTF* Time to treatment failure.

All patients harbored a *BRAF* V600E mutation. One patient (case #2) had an additional *BRAF* T599S mutation in exon 15. Seven of ten patients (70%) had somatic alterations of known significance in addition to *BRAF* mutations (Table 2). In addition, seven out of ten patients (70%) had somatic alterations of unclear significance (Additional file 3).

### Molecular alterations in responding patients

Two of three patients (66%) with long term CR (28.7+ and 23.6+ months) (cases #1 and 3) had a *BRAF* mutation as the only molecular alteration of known significance and one of seven patients (14%) with transient CR/PR had only a *BRAF* mutation (case #6). Alterations of known significance found in other patients included *NRAS* mutation (*n =* 2 patients), *PTEN* deletion (*n =* 1 patient), adenomatous polyposis coli (*APC*) mutation (*n =* 1 patient), cyclin-dependent kinase inhibitor *(CDKN)2A* or *2B* deletion (*n =* 3 patients), *PAX5* deletion (*n =* 1 patient), neurofibromin 1(*NF1*) mutation (*n =* 1 patient), *BRAF* amplification (*n =* 2 patients), *Aurora kinase A* amplification (*n =* 1), *MET* amplification (*n =* 1 patient), microphthalmia-associated transcription factor (MITF) amplification (*n =* 1 patient), and, v-myc avian myelocytomatosis viral oncogene homolog (*MYC*) amplification (*n =* 1 patient) (Table 2). New mutations within the *BRAF* gene itself were not observed except in one case (case #2) who attained a prolonged CR and had a T599S in addition to a V600E mutation.

### Responses in patients with simultaneous *NRAS* mutations on treatment

**Figure 3:**
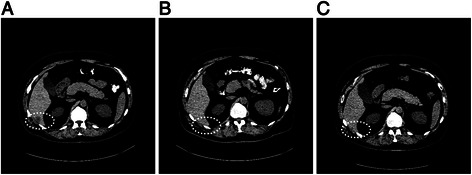
**Computed tomography scans. A)** at baseline, **B)** 2 months, and, **C)** 3.8 months after treatment initiation with a combination of a MEK and BRAF inhibitor, of a patient (case #8) with short-lived CR (TTF = 5.6 months), who demonstrated an *NRAS* mutation in a biopsy obtained 3.8 months after treatment initiation. At 2 months, the liver lesion has regressed; at 3.8 months it recurred.

*NRAS* mutations (Q61R and Q61K in codon 61) were detected in two of ten patients (20%). One patient (case #4) attained a PR (TTF = 11.2 months) and had an *NRAS* mutation on NGS analysis from tissue taken from a continuously progressing lesion 6 months after treatment initiation; adequate tissue sample for testing before treatment was not available. The other patient achieved a CR (case #8; TTF = 5.6 months) and had an *NRAS* mutation detected in a single PCR assay from a biopsy of the solitary hepatic lesion that progressed after a brief period of initial response (3.8 months after treatment initiation; Figure 3), while the pre-treatment NGS analysis on this patient failed to show an *NRAS* mutation (17 months before treatment).

### Molecular evolution with progression

Several patients had more than one biopsy performed, and molecular evolution was demonstrated in their results of molecular analysis. One patient with a CR (case #8; TTF = 5.6 months) had NGS analysis performed on a pre-treatment biopsy (17 months before treatment) that showed a *BRAF* and an *NF1* mutation. Subsequently, a second biopsy 3.8 months after treatment initiation showed an *NRAS* mutation (Q61K) by single PCR-based analysis (Table 2). One patient (case #5) had three different results on three different biopsy samples from progressive lesions (6, 9 and 26 months after treatment initiation) by NGS analysis. The first, at 6 months after treatment initiation, showed *CDKN2A, CDKN2B and PAX5* deletion (in addition to a *BRAF* mutation), while the second at 9 months after treatment started, showed an additional *NF1* truncation. A third NGS analysis obtained 26 months after treatment initiation demonstrated only alterations of unclear significance (Additional file 3). Of interest, this patient was having a mixed response to *BRAF*-targeted treatment at the time of the third biopsy. That biopsy, which no longer showed the *BRAF* mutation, was obtained from a tumor that was enlarging; other tumors that had shown the *BRAF* mutation were regressing on *BRAF*-targeted therapy.

### Timing of molecular analysis

One patient with an ongoing CR of 23.6+ months duration (case #3) had NGS analysis performed on a biopsy obtained 53 months prior to treatment; no alterations of known significance were demonstrated. A second biopsy performed 15 months prior to treatment showed a V600E *BRAF* mutation in a single PCR-based assay. This patient illustrates a phenomenon of interest, i.e., patients without *BRAF* mutations can acquire the mutation with progression, and they can be sensitive to cognate targeted inhibitors.

Another patient (case #6) with a biopsy 31 months prior to treatment had only a *BRAF* mutation among the alterations of known significance. This patient achieved a PR lasting 7.0 months. All other patients with biopsies performed 2 to 17 months prior to treatment had additional alterations of known significance, except for one individual (case #1) who had only a *BRAF* mutation and achieved a CR that is ongoing at 28.7+ months.

## Discussion

In order to better understand response and resistance, we analyzed patients with *BRAF* -mutant, advanced melanoma who achieved a PR or CR on a BRAF and/or MEK targeted agent. Previous reports [16] provide proof of principal for this next generation sequencing (NGS) platform. NGS identified a broad range of molecular alterations (Table 2; Additional file 3).

Most patients (*n =* 7) had multiple additional alterations of known oncogenic significance (Table 2). Pathways commonly affected, either directly or indirectly, included the PI3K/AKT/mammalian target of rapamycin (mTOR) axis (via *PTEN* alterations), the MAPK pathway (via *NRAS* mutations or *NF1* alterations), as well as tumor suppressor signals (via *CDKN2A* or *CDKN2B* deletion or *RB1* truncation). Other abnormalities included deletion in *PAX5* (a paired box transcription factor), and amplifications in *MET*, *BRAF* and *aurora kinase A*. The adenomatous polyposis coli (*APC*) gene responsible for familial adenomatous polyposis, was mutated in one patient; this gene, when aberrant, activates the Wnt signaling pathway and induces chromosomal instability [17,18]. Loss of its function also triggers the adenoma-carcinoma transition in colorectal cancer [19]. Additional mutations within the *BRAF* gene itself were not observed except for patient #2 who attained a prolonged CR and had a T599S in addition to a V600E mutation. *BRAF* amplification was seen in two patients.

These additional molecular abnormalities were at times evident long before treatment including, in one case, 17 months prior to starting targeted therapy (case #8). Only three patients (cases #1, 3 and 6) had no additional alterations of known significance as discerned by NGS, and two of these individuals achieved a prolonged CR with response ongoing 23.6+ months (case #3) and 28.7+ months (case #1), respectively, after starting treatment with a BRAF inhibitor. The third patient (case #6) achieved a transient PR (TTF = 7 months). In case #1, the tissue sample was obtained only 4 months prior to treatment. However, in cases 3 and 6, the tissue samples were acquired 53 and 31 months, respectively, prior to treatment; it is therefore unclear whether or not other biologically-significant alterations might have emerged closer to the time of treatment. One additional patient with a prolonged CR (TTF = 27.4+ months; case #2) demonstrated *BRAF* and *aurora kinase A* amplification in addition to a *BRAF* mutation in a tissue sample obtained 9 months before treatment.

Several observations herein warrant further exploration. First, it is conceivable that NGS analysis may reveal a subset of advanced disease that can achieve prolonged CR on a BRAF inhibitor alone. It is plausible that these patients are the ones that have only a *BRAF* alteration, or only alterations that influence a redundant signal. For most patients with melanoma, the emergence of resistance is expected. It is somewhat surprising that certain individuals can achieve a CR while harboring numerous potential driving alterations. For instance, the patient with a CR of 27.4+ months (case #2) had an NGS profile demonstrating several possible driver alterations (*BRAF* and *aurora kinase A* amplification) in addition to a *BRAF* mutation. Amplifications in these genes are known to confer resistance to treatment with BRAF inhibitors, though it has been suggested that the resistance driven by *BRAF* amplification can be overcome by higher doses of a BRAF inhibitor [20] or by combining MEK and BRAF inhibition [21]. The concept of oncogenic addiction may explain response in such individuals [22].

NGS analysis may also provide information that can be exploited to devise optimal combinations of targeted agents for patients at the time of their initial therapy or when resistance emerges. One patient who achieved a CR, albeit of short duration (TTF = 5.6 months; case #8), may have done so because he was treated with a combination of a MEK and BRAF inhibitor. NGS revealed a mutation in *NF1* in addition to a V600E *BRAF* mutation. *NF1* is an upstream suppressor that can modulate MEK signaling [23]. Such alterations confirm the need for strategies that incorporate combinations including BRAF and MEK inhibitors. In this patient, an *NRAS* mutation (Q61K) emerged 3.8 months after treatment initiation, which was demonstrated by a single PCR assay of the tissue sampled from the solitary liver lesion that progressed after treatment. This might have accounted for the patient’s relapse. The second patient (case #4) treated with a MEK and BRAF inhibitor combination demonstrated remarkable response (−34%; PR); a cystic lesion in the chest wall eventually showed progression. Fine needle aspiration cytology of the lesion revealed a Q61R mutation in the *NRAS* gene and other molecular aberrations in addition to the *BRAF* mutation.

Altogether two patients developed *NRAS* mutations after treatment (Figure 2) (cases #4 and #8). The emergence of an *NRAS* mutation is known to confer resistance to BRAF inhibitors [7], and may therefore explain the failure to achieve a durable response in these patients (n = 1 with PR and n =1 with transient CR) [24,25]. The presence of an *NRAS* mutation in addition to a *BRAF* mutation in patients treated with BRAF inhibitors may reactivate the MAP kinase (MAPK) pathway through CRAF [7]. Although MEK inhibitors might seem a rational choice for patients bearing *NRAS* alterations as anti-tumor activity has been reported in *NRAS*-mutant cutaneous melanoma patients on MEK 162 [26], experience suggests limited efficacy in such patients with other MEK inhibitors [27]. It is unclear whether this is due to the fact that NRAS signaling is incompletely extinguished by MEK inhibitors or if these tumors bear additional alterations that confer resistance.

One patient had a *PTEN* deletion (case #4). Alterations in the PI3K/AKT/mTOR pathway have been shown to be operative in multiple tumors including melanoma [28]. For instance, *PTEN* loss is found in 30-50% of melanomas [29], *PIK3CA* mutation in 3% [30], and changes in *AKT* expression in some melanomas [31]. These are actionable alterations in that multiple PIK3CA, AKT and mTOR inhibitors are in clinical trials or are already approved.

Recently, molecular evolution with progression and to some degree heterogeneity between tumors in individual patients has been described [32]. For instance, Wilmott and colleagues [33] reported different subclones in tumor tissue from a single metastatic site in a *BRAF*-mutant melanoma patient, following progression of disease after seven months of treatment with the BRAF inhibitor vemurafenib; one clone had an additional *NRAS* mutation. Our data supports such heterogeneity and demonstrates the role that advanced molecular technology may play in understanding and addressing mixed responses. For instance, a patient (case #5) with a PR for 7.9 months had three different biopsies from progressive lesions performed at 6, 9 and 26 months after treatment initiation. Each of these biopsies was analyzed using NGS and each demonstrated different results. The first two analyses, at 6 and 9 months, showed *CDKN2A, CDKN2B* and *PAX5* deletions, but the sample obtained 9 month after treatment initiation also showed an *NF1* truncation, probably indicating accumulation of additional changes with disease progression. The results from a sample obtained 26 months after treatment initiation, however, showed no *BRAF* mutation, or other molecular alterations of known significance. Of interest, the latter biopsy was taken from a tumor that was increasing in size on a BRAF inhibitor, while the other tumors had regressed.

There were several important limitations of this study. First, it is a retrospective study and as such tissue samples were not collected at a uniform time. Second the small number of patients in this pilot study precludes statistical assessments. Third, we focused on responders. Data on non-responders may be equally informative in identifying the mechanisms that confer resistance.

This study demonstrates multiple additional molecular alterations amongst *BRAF* mutation-positive patients with advanced melanoma who achieved a CR or PR on BRAF targeted agents. These alterations may be responsible for the development of resistance to treatment with BRAF and/or MEK inhibitors. We did not observe frequent new mutations in the *BRAF* gene itself correlating with resistance, consistent with the reported stability of the *BRAF* mutation in melanoma [34]; unlike the situation with other kinases such as *BCR-ABL* or epidermal growth factor receptor (EGFR), where resistance is often mediated by acquisition of additional mutations within the same gene. Given that some *BRAF*-mutant melanoma patients fail to respond to BRAF inhibitors, and that the majority of patients who do respond to BRAF/MEK inhibitors eventually develop resistance, there is an urgent need to identify possible combination treatments that may be effective. Of interest, some patients with advanced melanoma can achieve prolonged CR, and our investigation suggests that some of these individuals may harbor only a *BRAF* mutation despite the advanced state of their disease. Further, patients can achieve CR while still harboring aberrations other than those targeted by the agent given. (In our study, 2 of the 4 patients who achieved CRs harbored additional aberrations: *aurora kinase* and *BRAF* amplification in one patient, and an *NF1* and *NRAS* mutation in one patient). Therefore tumor complexity does not preclude a complete response. Finally, while obtaining biopsies close to the time of treatment may be ideal, our study illustrates that data obtained from biopsies distant to the time of treatment may still demonstrate aberrations that could be responsible for response and/or resistance. A concern that has been raised is whether patients with multiple aberrations can respond to targeted treatment of one of those aberrations, or whether such treatment is futile.

## Conclusion

Our study demonstrates that most of our excellent responders had multiple aberrations. Having these aberrations may (or may not) preclude “cure”, but they do not preclude an excellent response. Our study suggests that further investigation of NGS for identification of actionable molecular alterations before treatment and at the time of resistance is warranted.

## Additional files

Additional file 1:
**182 cancer-related genes and 37 introns of 14 genes involved in rearrangements sequenced by NGS.**

Additional file 2:
**Clinical trials and response assessment of 10 patients with** ***BRAF*** **mutation- positive melanoma.**

Additional file 3:
**Other NGS alterations of unclear significance** ^**a**^ **.**

## Abbreviations

PR: Partial response
CLIA: Certified molecular diagnostic laboratory
CR: Complete response
DNA: Deoxyribonucleic acid
ECOG: Eastern Cooperative Oncology Group
NGS: Next generation sequencing
PCR: Polymerase chain reaction
RECIST: Response evaluation criteria in solid tumors
TTF: Time to treatment failure
