# Supplementary material for: Next generation sequencing of exceptional responders with BRAF-mutant melanoma: implications for sensitivity and resistance
Source: BMC Cancer. 2015 Feb 18;15:61. doi: 10.1186/s12885-015-1029-z (PMC4340232; doi:10.1186/s12885-015-1029-z)
Supplement: Additional file 1: — 182 cancer-related genes and 37 introns of 14 genes involved in rearrangements sequenced by NGS. [file 12885_2015_1029_MOESM1_ESM.pdf]

Additional file 1. 182 cancer-related genes and 37 introns of 14 genes involved in rearrangements sequenced by NGS

ABL1	CDK6	FLT4	MEN1	PTPN11
ABL2	CDK8	FOXP4	MET	PTPRD
AKT1	CDKN2A	GATA1	MITF	PTPRD
AKT2	CDKN2B	GNA11	MLH1	RAF1
AKT3	CDKN2C	GNAQ	MLL	RARA
ALK	CEBPA	GNAS	MPL	RB1
APC	CHEK1	GPR124	MRE11A	RET
AR	CHEK2	GUCYIA2	MSH2	RICTOR
ARAF	CRKL	HOXA3	MSH6	RPTOR
ARFRP1	CRLF2	HRAS	MTOR	RUNX1
ARID1A	CTNNB1	HSP90AA1	MUTYH	SMAD2
ATM	DDR2	IDH1	MYC	SMAD4
ATR	DNMT3A	IDH2	MYCL1	SMARCA4
AURKA	DOT1L	IGF1R	MYCN	SMARCB1
AURKB	EGFR	IGF2R	NF1	SMO
BAP1	EPHA3	IKBKE	NF2	SOX10
BCL2	EPHA5	IKZF1	NKX2-1	SOX2
BCL2A1	EPHA6	INHBA	NOTCH1	SRC
BCL2L1	EPHA7	INSR	NPM1	STAT3
BCL2L2	EPHB1	IRS2	NRAS	STK11
BCL6	EPHB4	JAK1	NTRK1	SUFU
BRAF	EPHB6	JAK2	NTRK2	TBX22
BRCA1	ERBB2	JAK3	NTRK3	TET2
BRCA2	ERBB3	JUN	PAK3	TGFBR2
CARD11	ERBB4	KDM6A	PAX5	TNFAIP3
CBL	ERCC2	KDR	PDGFRA	TNKS
CCND1	ERG	KIT	PDGFRB	TNKS2
CCND2	ESR1	KRAS	PHLPP2	TOP1
CCND3	EZH2	LRP1B	PIK3CA	TP53
CCNE1	FANCA	LRP6	PIK3CG	TSC1
CD79A	FBXW7	LTK	PIK3R1	TSC2
CD79B	FGFR1	MAP2K1	PKHDI	USP9X
CDH1	FGFR2	MAP2K2	PLCG1	VHL
CDH2	FGFR3	MAP2K4	PRKDC	WT1
CDH20	FGFR4	MCL1	PTCH1	
CDH5	FLT1	MDM2	PTCH2	
CDK4	FLT3	MDM4	PTEN	

SELECT REARRANGEMENTS				
ALK	EGFR	ETV5	MLL	RET
BCR	ETV1	ETV6	RAF1	TMPRSS2
BRAF	ETV4	EWSR1	RARA	

Abbreviations: NGS, next generation sequencing
