# Supplementary material for: Next generation sequencing of exceptional responders with BRAF-mutant melanoma: implications for sensitivity and resistance
Source: BMC Cancer. 2015 Feb 18;15:61. doi: 10.1186/s12885-015-1029-z (PMC4340232; doi:10.1186/s12885-015-1029-z)
Supplement: Additional file 2: — Clinical trials and response assessment of 10 patients with BRAF mutation- positive melanoma. [file 12885_2015_1029_MOESM2_ESM.pdf]

Additional file 2. Clinical trials and response assessment of 10 patients with *BRAF* mutation- positive melanoma

Case No	Type of targeted drug	Clinical trial registry number	Response assessment
1	BRAF inhibitor	NCT00880321	RECIST 1.0
2	BRAF inhibitor	NCT00880321	RECIST 1.0
3	BRAF inhibitor	NCT00880321	RECIST 1.0
4	BRAF + MEK inhibitors	NCT01072175	RECIST 1.1
5	BRAF inhibitor	NCT00405587	RECIST 1.0
6	MEK inhibitor	NCT00687622	RECIST 1.0
7	BRAF inhibitor	NCT00880321	RECIST 1.0
8	BRAF + MEK inhibitors	NCT01072175	RECIST 1.1
9	BRAF inhibitor	NCT00880321	RECIST 1.0
10	BRAF inhibitor	NCT00880321	RECIST 1.0

Abbreviations: RECIST, Response Evaluation Criteria in Solid Tumors
