# Supplementary material for: Next generation sequencing of exceptional responders with BRAF-mutant melanoma: implications for sensitivity and resistance
Source: BMC Cancer. 2015 Feb 18;15:61. doi: 10.1186/s12885-015-1029-z (PMC4340232; doi:10.1186/s12885-015-1029-z)
Supplement: Additional file 3: — Other NGS alterations of unclear significance a . [file 12885_2015_1029_MOESM3_ESM.pdf]

Additional file 3. Other NGS alterations of unclear significance[a]

Case No.	Date of sample	Other NGS alterations
1	5/1/2009	BCL2:NM_000633:c.176C>T_p.P59L(0.13,1195),<br>EGFR:NM_005228:c.3407C>T_p.P1136L(0.32,1661),<br>GUCY1A2:NM_000855:c.1792G>A_p.E598K(0.14,1271),<br>HOXA3:NM_153631:c.1188G>A_p.M396I(0.07,843),<br>PKHD1:NM_138694:c.6854G>A_p.G2285E(0.50,1303)
2	1/22/2009	
3	8/19/2005	DOT1L:NM_032482:c.4327G>T_p.G1443C(0.51,682)
4	5/17/2011	
5	12/2/2009	DNMT3A:NM_022552:c.89A>C_p.E30A(0.45,392),<br>EZH2:NM_004456:c.1628T>A_p.I543K(0.23,878),<br>EZH2:NM_004456:c.1638T>A_p.N546K(0.24,904),<br>HSP90AA1:NM_001017963:c.1093_1094insTTTCTT:nonframeshift(0.34,1114),<br>MTOR:NM_004958:c.985G>A_p.A329T(0.47,544),
	3/23/2010	DNMT3A:NM_022552:c.89A>C_p.E30A(0.48,415),<br>HSP90AA1:NM_001017963:c.1093_1094insTTTCTT:nonframeshift(0.34,528),<br>MTOR:NM_004958:c.985G>A_p.A329T(0.48,543),
	8/24/2011	APC:NM_000038:c.560G>A_p.R187K(0.05,799),<br>HSP90AA1:NM_001017963:c.1093_1094insTTTCTT:nonframeshift(0.37,803),<br>NOTCH1:NM_017617:c.1154C>T_p.S385F(0.06,374),<br>NTRK3:NM_001007156:c.1748T>A_p.I583K(0.05,993),<br>RET:NM_020975:c.2971G>A_p.E991K(0.05,457)
6	9/17/2007	PTPRD:NM_002839:c.1292C>T_p.S431L(0.07,1289)
7	11/5/2009	BCL6:NM_001706:c.492G>T_p.E164D(0.52,212),<br>ERBB2:NM_004448:c.1573C>T_p.P525S(0.21,154),<br>ERBB3:NM_001982:c.1423C>T_p.R475W(0.37,348),<br>LRP1B:NM_018557:c.2369G>A_p.R790Q(0.13,293),<br>MAP2K2:NM_030662:c.416G>A_p.S139N(0.24,294)
8	4/7/2009	

9	11/11/2009	EPHA7:NM_004440:c.1141C>T_p.P381S(0.25,1240), JAK3:NM_000215:c.2675G>A_p.G892D(0.21,914),PKHD1:NM_138694:c.8314C>T_p.L2772F(0.27,1001), PRDM12:NM_021619:570+1delGT:splice(0.14,37), PRKDC:NM_006904:c.6479C>T_p.T2160M(0.38,1114), SMARCA4:NM_003072:c.3595G>A_p.V1199M(0.29,375)
10	12/11/2009	BRCA1:NM_007294:c.1888A>C_p.N630H(0.24,1044), BRCA2:NM_000059:c.8709G>T_p.E2903D(0.20,953), FCGR3A:NM_001127593:c.577+1C>T:splice(0.35,293), FLT3:NM_004119:c.2323G>A_p.E775K(0.21,1467), LTK:NM_002344:c.680C>T_p.P227L(0.14,570), MPL:NM_005373:c.1033C>A_p.Q345K(0.21,524), MRE11A:NM_005590:c.1526_1526delT_p.N511fs*13:frameshift(0.20,1186), PHLPP2:NM_015020:c.1532+1C>T:splice(0.24,815), TET2:NM_001127208:c.2087C>T_p.S696F(0.39,1119)

Abbreviations: NGS, next generation sequencing

[a]Molecular alterations that are of unclear significance refer to specific alterations whose functional impact is not known at present to impact tumorigenesis.
